# Identification of risk and health budget

**DOI:** 10.1186/1824-7288-40-S1-A65

**Published:** 2014-08-11

**Authors:** Antonina Lo Cascio

**Affiliations:** 1 Pediatra di Famiglia Palermo, Docente Scuola Specializzazione Pediatria Università di Palermo, Italy

## 

The Language is a specific competence of human species that develops in a relatively short period of the life. Indeed, most of the linguistic rules are learned by the child within 4-5 years of life. In his firsts months of life, through the interaction with adults, the baby acquires the first signs of communication through the eye, sounds, facial expression, and response to the environmental stimulus that surrounds him.

Assessment of health status ("Bilancio di Salute"), regularly carried out by the primary care Pediatricians, are used to carry out surveillance longitudinal neuro- evolutionary development of the child through the integration of its communicative and linguistic, auditory, motor and visual-spatial skills. Since the sixth month of the child’s life is possible to detect the risk indicators that deserve a very early assessment, as long as it has been excluded, through screening with otoacoustic emissions effected in birth centers, a hearing disorder.

In the first three years of life, the presence of an atypical development of language should be underlined by Pediatricians and parents, because it could not be just a temporary condition related to individual variability.

The “wait and see” approach can strongly affect the social life and learning at school.

The age of three is a sort of watershed between the so-called “late talkers” and children with a specific language disorder, provided that the child understands the language of the adult. Lowering the age of first consultation is recommended for increase the possibility of early detection, and then an early rehabilitative intervention, leading to an improvement in the prognosis of the pathology [1]. A delay in the acquisition of language can be an indicator of mild intellectual disability, that is often recognized only after the beginning of school activities.

An article published in May 2014 [2] aims to evaluate the association between the pragmatic skills of language and behavioral problems in a group of 40 adolescents who had experienced childhood speech problems. Longitudinally evaluation at the age of 7-9 years (T1) and at the age of 12-15 years (T2) was made with a control group (37) of typically developing.

The results obtained prove that the atypical group shows a deficit of linguistic, and that emotional problems , language and difficulties with peers at 7-9 years are closely related with the pragmatic difficulties in adolescence, and that they affect in a negative way social relations.
